# Etomidate ameliorated advanced glycation end-products (AGEs)-induced reduction of extracellular matrix genes expression in chondrocytes

**DOI:** 10.1080/21655979.2021.1951926

**Published:** 2021-07-24

**Authors:** Xiaohua Sun, Jizheng Zhang, Yi Li, Wanlu Ren, Lijun Wang

**Affiliations:** a Department of Anesthesiology, Outpatient and Emergency, Tianjin Hospital, Tianjin, China; b Department of Anus& Intestine Surgery, Tianjin Hospital, Tianjin, China

**Keywords:** Etomidate, osteoarthritis, AGEs, ECM, SOX-9

## Abstract

Osteoarthritis (OA) is a rheumatic disease common in the elderly. AGEs are the end products of glycation reactions and play an important role in the development of OA. Etomidate is a general anesthesia-inducing agent recently reported to exert significant anti-inflammatory effects. The present study aims to explore the protective effect of Etomidate against advanced glycation end-products (AGEs)-induced reduction of extracellular matrix gene expression in chondrocytes. In the present study, we found that AGEs significantly reduced the expression of Collagen II (*COL2A1*) and Aggrecan (*ACAN*) at the gene level. Furthermore, AGEs inhibited the expression of SRY-related high mobility group-box gene 9 (SOX-9), promoting the expression of COL2A1 and ACAN. *COL2A1, ACAN*, and *SOX-9* in chondrocytes were significantly elevated by treatment with Etomidate alone. Consistently, Etomidate ameliorated AGEs-induced downregulation of *COL2A1, ACAN*, and SOX-9 in a dose-dependent manner. Importantly, we found that knockdown of SOX-9 eliminated the beneficial effects of Etomidate against AGEs-induced decrease in *COL2A1 and ACAN genes*. Based on these findings, we demonstrated that Etomidate could ameliorate AGEs-induced reduction of extracellular matrix gene expression in chondrocytes by upregulating SOX-9.

## Graphical abstract

**Figure:**
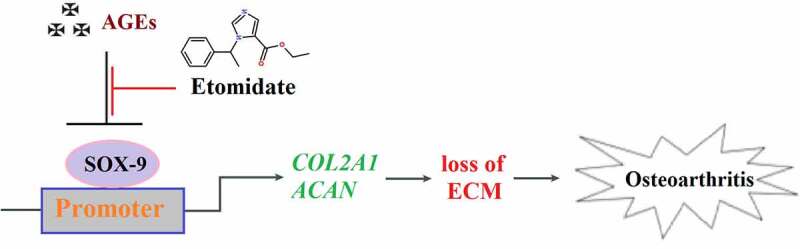

## Introduction

Osteoarthritis (OA) is a rheumatic disease with high morbidity in people over 60 years of age [1]. It is accompanied by knee or hip joint pain and dysfunction and is currently lacking effective clinical therapies [2,3]. OA is considered as a disease of the entire joint, affecting all joint tissue. Thus, it is characterized not only by the degeneration of the cartilage and inflammation of the synovial membrane but also by meniscal degeneration, subchondral sclerosis, inflammation, and fibrosis of the infrapatellar fat pad [4]. However, the most significant pathological characteristic of OA is the degenerative disruption of cartilage tissues. It is widely accepted that maintaining normal metabolism and preventing the destruction of cartilage homeostasis are key steps in the treatment of OA [5,6].

As the main cell type of cartilage tissues, chondrocytes are responsible for the synthesis of extracellular matrix (ECM) and provide sufficient mechanical support for the shear force and pressure produced from articular movements [7,8]. ECM is mainly composed of collagens, proteoglycan, structural aggrecans, lipids, and water. A grid structure is formed by the arranged collagens to maintain the morphology and rigidity of cartilage tissues. Collagen II is the main subtype of collagens and is encoded by collagen type II alpha-1 gene (*COL2A1*) [9]. It is reported that the degradation of collagen II is observed in OA cartilage, and the fragments of collagen fibronectin produced from the degraded collagen II aggravate the further degradation of cartilage tissues.

Advanced glycation end-products (AGEs) are the end products of glycation reactions. It has been reported that increased accumulation of AGEs in cartilage plays an important role in the pathogenesis of OA [10]. The interaction of AGEs with receptor for AGEs (RAGE) is responsible for the inflammatory response in the development of OA through the upregulation of pro-inflammatory cytokines, which further stimulates the expression of matrix metalloproteinases (MMPs) and disintegration and metalloproteinase with thrombospondin motifs (ADAMTS) [11].

**Figure 1.**
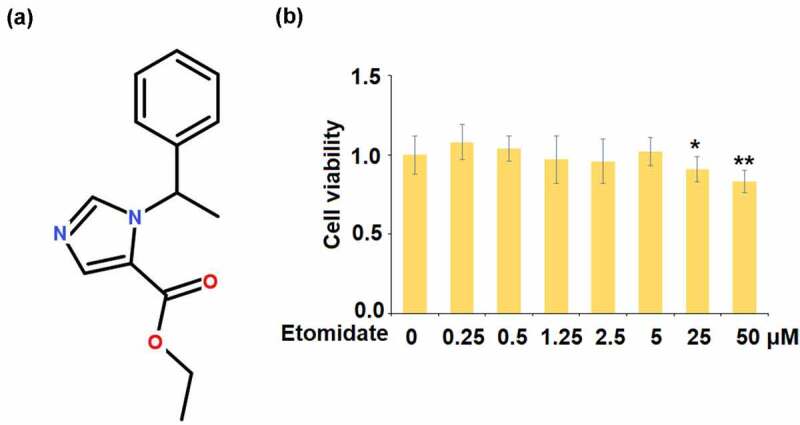
Effects of Etomidate in cell viability. (a). Molecular structure of Etomidate; (b). Human SW1353 chondrocytes were stimulated with Etomidate alone at 0.25, 0.5, 1.25, 2.5, 5, 25, and 50 μM for 24 hours, cell viability was measured using the MTT assay (*. **, P < 0.05, 0.01 vs. control, N = 5–6)

Aggrecan is another main component of ECM and the decomposition of aggrecan induced by elevated expressions of MMPs and ADAMTS, is involved in the pathogenesis of OA. SOX-9 is an important transcriptional factor involved in the regulation of developing cartilage [12], reported to be significantly downregulated in the OA cartilage tissues [13]. In the development and processing of cartilage, a significant parallel correlation is observed on *SOX-9* and *COL2A1* [14]. Bel claimed that the expression of *COL2A1* in cartilage tissues was directly regulated by SOX-9 through binding with the promoter of *COL2A1* [15], which is further confirmed by the investigation in TNF-α treated chondrocytes reported by Wang [16]. Hence, activating the function of SOX-9 may be a useful way for treating OA. Etomidate (Figure 1a) is a general anesthesia inducer developed by Janssen Pharmaceuticals [17] and approved for clinical trials by the U.S Food and Drug Administration in 1972 [18]. Etomidate is a highly selective agonist of γ-aminobutyric acid type A receptors (GABBAARs) and exerts pharmacological effects by enhancing the affinity between the inhibitory neurotransmitter GABA and the GABBAARs. Recently, promising anti-inhibitory effects of Etomidate have been widely reported. Liu reported that the production of pro-inflammatory cytokines in LPS-treated rat macrophages was significantly mitigated by Etomidate. Etomidate, according to Li, protects against ischemia-reperfusion injury by lowering inflammatory markers. However, whether Etomidate has any beneficial capacity in chondrocytes and the pathogenesis of OA still needs to be elucidated. In this study, we aim to investigate the protective effect of Etomidate against AGEs-induced insults in chondrocytes and clarify the underlying mechanism.

## Materials and methods

### Cell culture and transfection

SW1353 chondrocytes were obtained from ATCC (ATCC, Manassas, USA) and cultured in DMEM supplemented with 5% FBS at 37°C and 5% CO_2_ [19]. A specific siRNA was designed and transfected into human SW1353 chondrocytes together with the transfection agent lipofectamine 3000 (Thermo, MA, USA) according to the instructions of the manufacturer, followed by verifying the efficacy of silencing SOX-9 using Western blotting assay. Cells were stimulated with 1.25, 2.5, and 5 μM Etomidate (#E6530, Sigma-Aldrich, USA) for 24 hours [20]. Etomidate was dissolved in DMSO. Cells were then stimulated with 100 μg/mL AGEs (Glycosylated BSA, # bs-1158P, Beijing Biosynthesis Biotechnology, China).

### Cell viability determination

Human SW1353 chondrocytes were stimulated with Etomidate alone at 0.25, 0.5, 1.25, 2.5, 5, 25, and 50 μM for 24 hours. After treatment, media was discarded from cell cultures. 50 µL of serum-free media and 50 µL of 3-(4,5)-dimethylthiahiazo (-z-y1)-3,5-di- phenytetrazoliumromide (MTT) solution were added into each well. After incubation for 37°C for 3 hours, 150 µL DMSO was added to dissolve the MTT formazan by wrapping the plate in foil and shaking it on an orbital shaker for 15 minutes. Absorbance was read at 590 nM to calculate cell viability.

### Real-time PCR analysis

After isolating the total RNA from the treated human SW1353 chondrocytes using the TRIzol reagent (Invitrogen, California, USA), the RNAs were quantified and transcribed into cDNA utilizing the Revert Aid First-strand cDNA Synthesis Kit (Thermo, Massachusetts, USA) based on the manufacturers’ instruction. Then, the SYBR system (Invitrogen, California, USA) was used to perform the real-time PCR in a 20 μL PCR reaction system. Lastly, the 2^−ΔΔCt^ method was applied to calculate the expression of target genes, with GAPDH as a normalization gene [21]. The primer sequences are listed in Table 1.

**Table 1.** Primer sequences

Genes	Forward (5ʹ-3ʹ)	Reverse (5‘-3ʹ)
SOX9	5ʹ-AGACTCACATCTCTCCTAATGCT-3’	5ʹ-ACGTCGGTTTTGGGAGTGG-3’
COL2A1	5ʹ-TTCCTCCGTCTACTGTCCAC-3’	5ʹ-ACATCATTGGAGCCCTGGAT-3’
ACAN	5ʹ-CCTGATGTAAGCGAAGCCA-3’	5ʹ-CTGGTGCTGACTCATCTGT-3’
GAPDH	5ʹ-ACTGGCGTCTTCACCACCAT −3’	5ʹ-AAGGCCATGCCAGTGAGCTT −3’

### Western blot assay

After lysing the treated human SW1353 chondrocytes with the cell lysis buffer (Invitrogen, California, USA), the total proteins were extracted for quantification using a BCA kit (Beyotime, Shanghai, China) and were further loaded and run on the SDS-PAGE. Subsequently, the proteins were transferred onto the PVDF membrane, followed by being blocked with 5% nonfat milk. After washing 3 times, the membrane was incubated with primary antibodies against SOX-9 (1:1000, CST, Boston, USA) and GAPDH (1:1000, CST, Boston, USA) at 4°C overnight, followed by being incubation with secondary antibody. Lastly, the blots were incubated with ECL solution and exposed to the Tonan exposure (Tonan, Shanghai, China). The blots were further quantified using the Image J software [22].

### Immunofluorescence staining

After washing the human SW1353 chondrocytes with PBS buffer, cells were fixed using 4% paraformaldehyde (Sigma-Aldrich, USA) and permeabilized with 0.1% Triton X-100 (Sigma-Aldrich, USA), followed by being incubation with the primary antibody against SOX-9 (ab185966 Abcam, Cambridge, UK) at room temperature for 2 hours. Subsequently, the appropriate secondary antibodies conjugated with TRITC and DAPI (a nuclear marker and the color is blue) were used to incubate with the collected cells. Lastly, the fluorescence density of each group was observed under a fluorescence microscope (Olympus, Toyko, Japan) [23].

### Statistical analysis

All experiments were repeated at least three times. All data were expressed as the mean ± SD and analyzed by ANOVA, followed by Turkey’s post-hoc test, using Graph Pad Prism 6.0. P less than 0.05 was considered significant [24].

## Results

Using an *in vitro* SW1353 chondrocytes model stimulated with AGEs, we examined the function of Etomidate in the expression of matrix biomarkers. We found that Etomidate mitigated AGEs-induced reduction of *COL2A1* and *ACAN* by increasing the expression of the transcriptional factor SOX-9.

## Etomidate promoted *COL2A1, ACAN* in human SW1353 chondrocytes

To screen the optimized concentrations of Etomidate for cell culture, human SW1353 chondrocytes were stimulated with Etomidate alone at 0.25, 0.5, 1.25, 2.5, 5, 25, and 50 μM, cell viability was measured using the MTT assay. Interestingly, we found that when the concentration of Etomidate was lower than 5 μM, it did not affect cell viability. However, exposure to 25, and 50 μM Etomidate resulted in lower cell viability of human SW1353 chondrocytes (Figure 1b). Therefore, 1.25, 2.5, and 5 μM Etomidate were used in the subsequent experiments.

**Figure 2.**
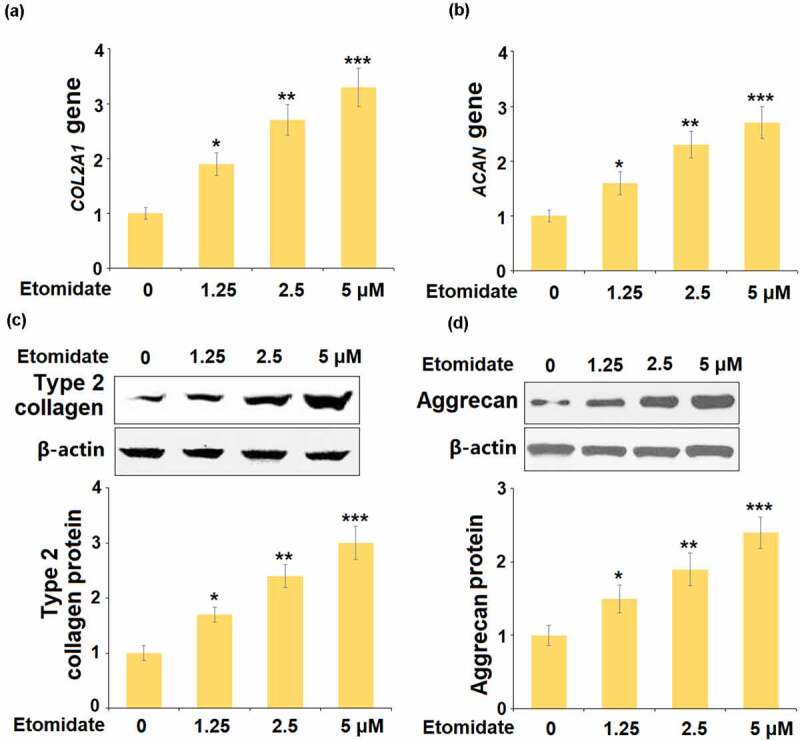
Etomidate promoted the expression of *COL2A1* and *ACAN*. Cells were stimulated with 1.25, 2.5, and 5 μM Etomidate for 24 hours. (a). mRNA of *COL2A1;* (b). mRNA of *ACAN;* (c). Protein expression of type 2 collagen; (d). Protein expression of Aggrecan (*. **, ***, P < 0.05, 0.01, 0.005 vs. control, N = 5–6)

To assess the roles of Etomidate on the components of the ECM, cells were stimulated with 1.25, 2.5, and 5 μM Etomidate for 24 hours. The levels of *COL2A1* and *ACAN* were significantly elevated by the presence of Etomidate (Figure 2a and Figure 2b). Consistently, we found that treatment with Etomidate increased the protein expressions of type 2 collagen (Figure 2c) and aggrecan (Figure 2d) in a dose-dependent manner.

## Etomidate upregulated SOX-9

**Figure 3.**
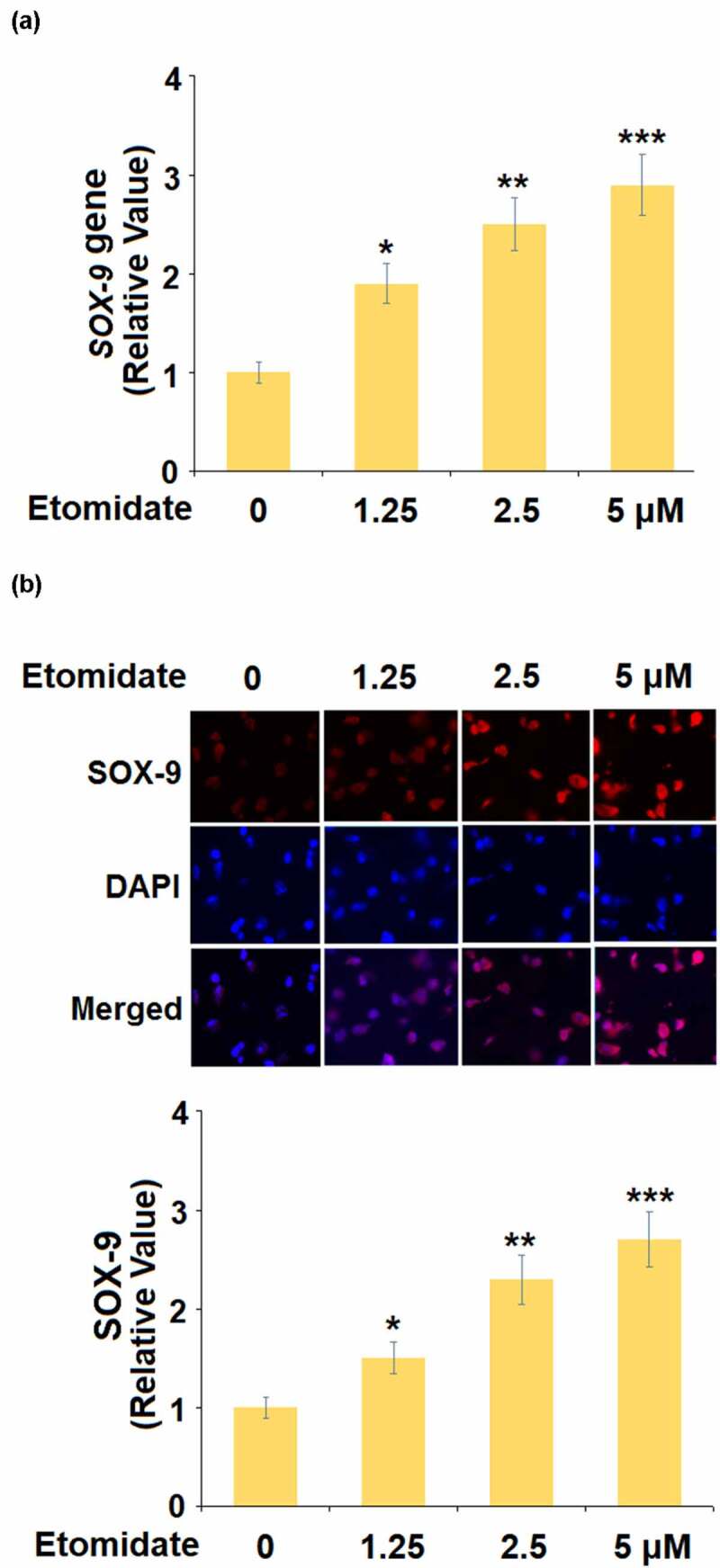
Etomidate upregulated SOX-9. Cells were stimulated with 1.25, 2.5, and 5 μM Etomidate for 24 hours. (a). mRNA of SOX-9; (b). Protein of SOX-9 (*. **, ***, P < 0.05, 0.01, 0.005 vs. control group, N = 6)

To evaluate the potential mechanism underlying the effect of Etomidate on *COL2A1* and *ACAN*, the expression of SOX-9 was detected following stimulation with 1.25, 2.5, 5 μM Etomidate for 24 hours. As shown in Figure 3, as the concentration of Etomidate increased from 1.25 to 5 μM, the expression of SOX-9 was dramatically promoted by treatment with Etomidate.

## Etomidate ameliorated AGEs-induced decrease in the expression of *COL2A1 and ACAN*

**Figure 4.**
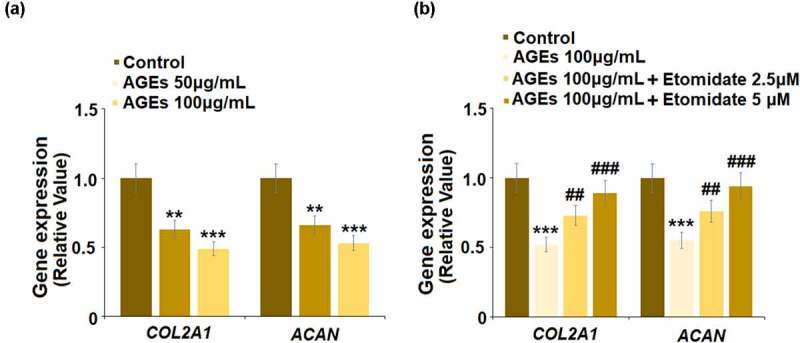
Etomidate ameliorated AGEs-induced decrease in *COL2A1 and ACAN*. (a). Cells were stimulated with AGEs (50, and 100 μg/mL) for 24 hours. Gene levels of *COL2A1* and *ACAN* were measured; (b). Cells were stimulated with AGEs (100 μg/mL) with 2.5, and 5 μM Etomidate for 24 hours. Gene levels of *COL2A1* and *ACAN* were measured (**, ***, P < 0.01, 0.005 vs. control group; ##, ###, P < 0.01, 0.005 vs. AGEs treatment group, N = 6)

To evaluate the protective effect of Etomidate on ECM degradation induced by AGEs, cells were stimulated with AGEs (50, and 100 μg/mL) for 24 hours. As shown in Figure 4a, *COL2A1* and *ACAN* were significantly inhibited by stimulation with AGEs, indicating that the *in-vitro* model was successfully established. Subsequently, cells were stimulated with AGEs (100 μg/mL) with Etomidate. Compared to the control group, *COL2A1* and *ACAN* were reduced by AGEs, which were upregulated by treatment with Etomidate, suggesting a beneficial effect of Etomidate on ECM degradation induced by AGEs.

To further confirm the beneficial effects of Etomidate on the expression of *COL2A1* and *ACAN*, human C28/I2 chondrocytes cell lines were used. We found that exposure to AGEs reduced the expression of both *COL2A1* and *ACAN* in human C28/I2 chondrocytes, which were rescued by Etomidate in a dose-dependent manner (Supplementary Figure S1).

## Etomidate ameliorated AGEs-induced reduction of SOX-9

**Figure 5.**
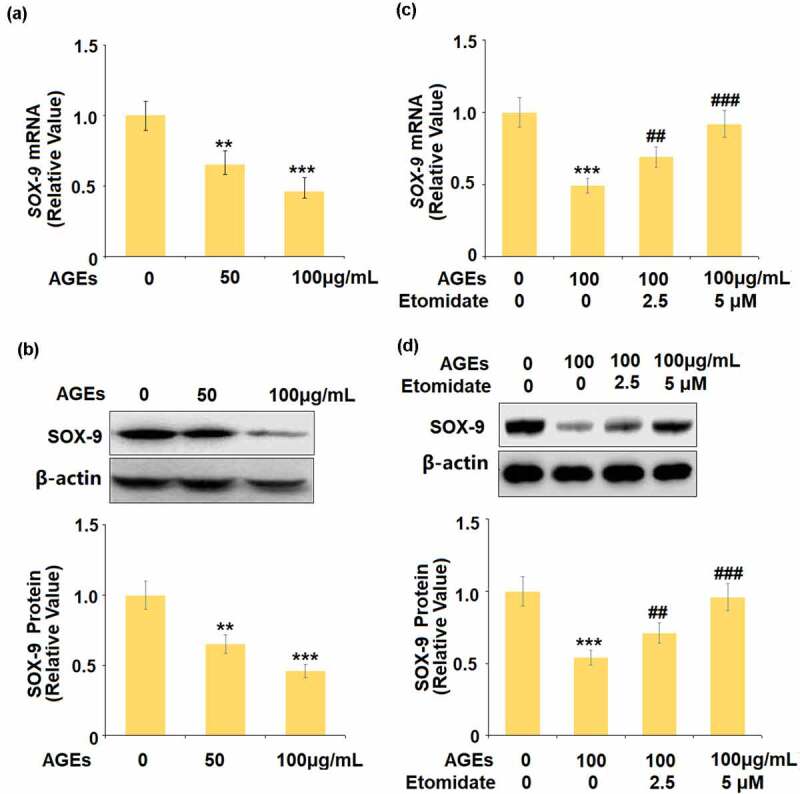
Etomidate ameliorated AGEs-induced reduction of SOX-9. (a-b). Cells were stimulated with AGEs (50, and 100 μg/mL) for 24 hours. mRNA and protein levels of SOX-9; (c-d). Cells were stimulated with AGEs (100 μg/mL) with 2.5, and 5 μM Etomidate for 24 hours. Expression of SOX-9 were measured (**, ***, P < 0.01, 0.005 vs. vehicle group; ##, ###, P < 0.01, 0.005 vs. AGEs treatment group, N = 5)

We firstly evaluated the effects of Etomidate on SOX-9 in chondrocytes. The gene and protein expression levels of SOX-9 were significantly suppressed by the stimulation with AGEs (Figure 5a and Figure 5b). In the presence of Etomidate, the downregulated gene and protein expression levels of SOX-9 caused by AGEs were remarkably alleviated by Etomidate.

### *Knockdown of SOX-9 abolished the effects of Etomidate against AGEs-induced reduction of* COL2A1 *and* ACAN *genes*

**Figure 6.**
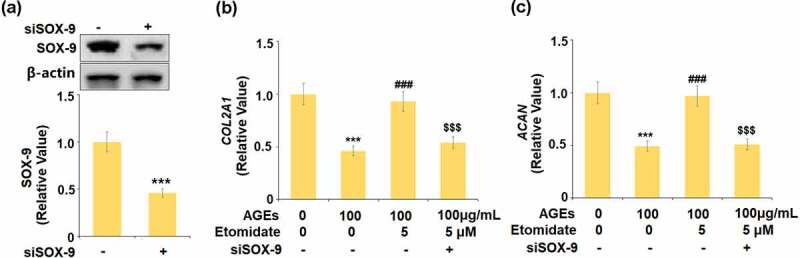
Knockdown of SOX-9 abolished the beneficial effects of Etomidate against AGEs-induced reduction of *COL2A1 and ACAN* genes. Cells were transfected with siRNA SOX-9, followed by stimulation with AGEs (100 μg/mL) with 5 μM Etomidate for 24 hours. (a). Successful knockdown of SOX-9; (b). Gene level of *COL2A1; (c)*. Gene level *of ACAN* (***, P < 0.005 vs. vehicle group; ###, P < 0.005 vs. AGEs treatment group; $$$, P < 0.005 vs. AGEs+ Etomidate group, N = 5–6)

To further confirm that Etomidate protected the degradation of ECM through upregulating SOX-9, cells were transfected with SOX-9 siRNA. The expression of SOX-9 was significantly downregulated in the siRNA transfected cells, indicating that the SOX-9 silenced chondrocytes were successfully established (Figure 6a). *COL2A1* and *ACAN* were remarkably reduced in AGEs-challenged cells and pronouncedly elevated by treatment with Etomidate. However, the knockdown of SOX-9 abolished the effects of Etomidate against AGEs-induced reduction of *COL2A1* and *ACAN*. These data indicate that Etomidate might protect the reduction of ECM genes through upregulating SOX-9.

## Discussion

The development of OA is closely related to factors such as aging, heredity, development, metabolism, and trauma, with aging being one of the most important variables. Excessive development and buildup of advanced glycation end products (AGEs) are commonly generated as people get older [25,26].

AGEs are the stable covalent compounds derived from the non-enzymatic glycation between proteins or lipids and glucose or other reductive monosaccharides, which is an inescapable and irreversible process in our body. It is reported that compared to other tissues, AGEs are highly expressed on cartilage tissues and are closely associated with the pathogenesis of OA [27]. The collagen injury and development of OA symptoms can be directly induced by upregulating AGEs, which are reported to be alleviated by the inhibitor of AGEs formation or crosslinking [28,29]. Although the pathological mechanism underlying the inducement of AGEs for OA, is unknown, it has been proven that stimulation of AGEs causes glycosylation of articular cartilage collagen and a decrease in aggrecan synthesis which further contributes to the increase of matrix brittleness, and a decrease in resistance to pressure or shear force [30,31]. In the present study, AGEs were used to simulate the *in vitro* injury model on chondrocytes, which was verified by the downregulation of *COL2A1* and *ACAN*, indicating that the ECM degradation might be induced by the stimulation with AGEs.

After Etomidate treatment, *COL2A1* and *ACAN* were dramatically elevated, showing that Etomidate could help to restore the degraded ECM caused by AGEs. In our future work, more elements will be taken into consideration to better understand the protective effect of Etomidate against ECM degradation, including measuring the expression of MMPs and detecting the production of inflammatory factors, which are reported to be involved in the pathological changes in chondrocytes induced by AGEs [31]. Furthermore, the therapeutic effect of Etomidate against OA will be further confirmed in animal models.

SOX-9 is widely expressed in precursor cells of cartilage and mature chondrocytes. It is reported that it directly regulates the transcription process of *COL2Α1* by targeting specific binding sites located in the intron of *COL2Α1* [32]. SOX-9 not only impacts the formation and differentiation of cartilage but also promotes the differentiation of medullary mesenchymal stem cells into chondrocytes. Lee [33] claimed that SOX-9, along with SOX-5 and SOX-6, participate in the repairment of chondrocytes by promoting the differentiation from medullary mesenchymal stem cells into chondrocytes. It is reported that the expression of ECM components was significantly elevated by transfecting the *SOX-9* gene into the chondrocytes [34]. In addition, SOX-9 is the upstream gene of Col18α1 and Col12α1 and regulates the expression of these proteins [35,36]. In the present study, we reported that SOX-9 was significantly suppressed by the stimulation with AGEs, revealing a potential pathological mechanism underlying the effects of AGEs on chondrocytes, which will be further verified in the animal model in our future work. Through the treatment with Etomidate, the downregulated SOX-9 was significantly reversed, indicating that the protective effects of Etomidate on degraded ECM components may be related to the upregulation of SOX-9. We further established the SOX-9 knockdown chondrocytes to verify the hypothesis. In our future work, the regulatory effect of Etomidate on SOX-9 will be further proved in the animal model to provide sufficient evidence for the application of Etomidate in the clinical treatment of OA.

Although the pathological mechanism of OA is still complex, it is well known that the inflammatory response plays a critical role in the progression of OA [37]. Pro-inflammatory cytokines, such as interleukin-1β (IL-1β) and tumor necrosis factor-α (TNF-α), could activate many inflammatory signaling pathways including nuclear factor – κB (NF-κB) to promote an inflammatory cascade. It is well known that RAGE, a member of the immunoglobulin superfamily, is expressed in human chondrocytes [38]. When AGEs bind to RAGE, they activate MAP kinase signaling, resulting in upregulation of NF-κB activity, which promotes the inflammatory cascades [39]. Furthermore, activated NF-κB can enhance the expressions of MMPs and ADAMTS, leading to cartilage degradation [40,41]. Previous studies have demonstrated the anti-inflammatory properties of Etomidate by inhibiting the NF-κB activity and suppressing the expression of pro-inflammatory cytokines [10]. Consistently, our results indicate that the presence of Etomidate not only inhibits AGEs-induced expressions of IL-1β and TNF-α, but also the expressions of MMP-3, MMP-13, ADAMTS-4, and ADAMTS-5 (Data not shown). Our future investigations will provide a complete picture of the underlying mechanism through which Etomidate exerts its protective attributes in OA.

There are several limitations of the current study. Firstly, the major findings here are based on *in vitro* cell culture studies. It should be noted that the pathological mechanism of OA is complicated and various risk factors have been involved [42]. In vivo studies with appropriate animal models are necessary to further confirm the protective effect of etomidate in future. Secondly, it has been recently reported that administration of etomidate at a high dose will have an increased risk for adverse effects in the cardiovascular, respiratory, and central nervous systems [43].

## Conclusion

In conclusion, our data revealed that Etomidate might rescue the AGEs-induced downregulation of *COL2A1* and *ACAN* to prevent reduction of extracellular matrix genes in chondrocytes by upregulating SOX-9.

## Supplementary Material

Supplemental Material

## Data Availability

Data are available on the reasonable request to the corresponding author.
