# Supplementary figures and images for: Etomidate ameliorated advanced glycation end-products (AGEs)-induced reduction of extracellular matrix genes expression in chondrocytes
Source: Bioengineered. 2021 Jul 24;12(1):4191–200. doi: 10.1080/21655979.2021.1951926 (PMC8806553; doi:10.1080/21655979.2021.1951926)

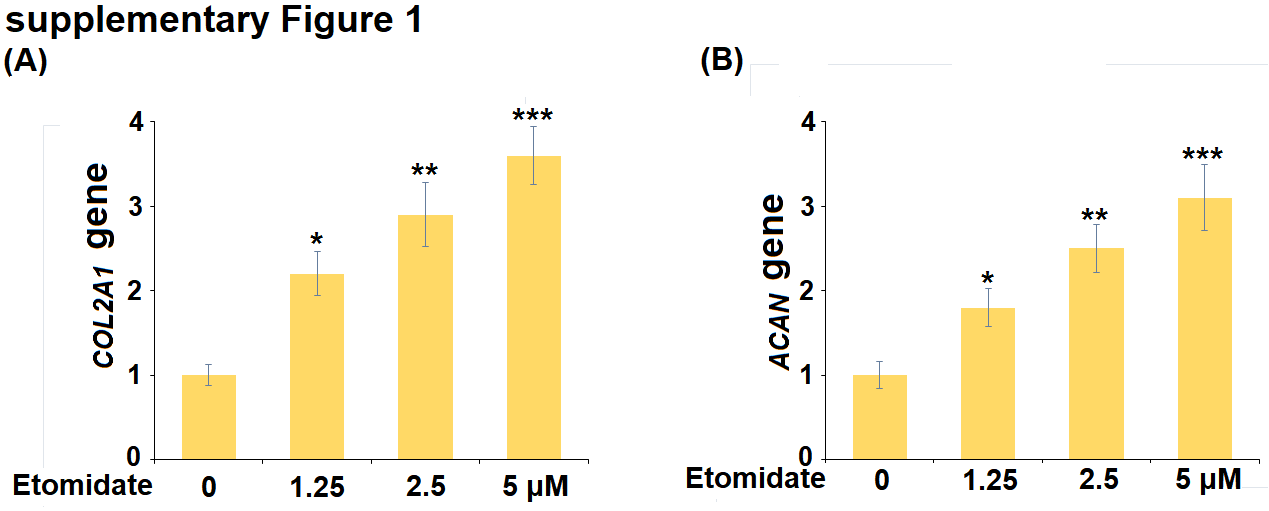

Supplement: Supplemental Material [file KBIE_A_1951926_SM8471.zip › supplementary Figure 1.tif]
